# Outcomes Following Early Postoperative Adjuvant Radiosurgery for Brain Metastases

**DOI:** 10.1001/jamanetworkopen.2023.40654

**Published:** 2023-10-31

**Authors:** Evan D. Bander, Tarek Y. El Ahmadieh, Justin Chen, Anne S. Reiner, Samantha Brown, Alexandra M. Giantini-Larsen, Robert J. Young, Kathryn Beal, Brandon S. Imber, Luke R. G. Pike, Cameron W. Brennan, Viviane Tabar, Katherine S. Panageas, Nelson S. Moss

**Affiliations:** 1 Department of Neurosurgery and Brain Metastasis Center, Memorial Sloan Kettering Cancer Center, New York, New York; 2 Department of Neurosurgery, New York Presbyterian Hospital/Weill Cornell Medical College, New York; 3 Department of Neurosurgery, Loma Linda University Health, Loma Linda, California; 4 Department of Epidemiology & Biostatistics, Memorial Sloan Kettering Cancer Center, New York, New York; 5 Department of Radiology, Memorial Sloan Kettering Cancer Center, New York, New York; 6 Department of Radiation Oncology and Brain Metastasis Center, Memorial Sloan Kettering Cancer Center, New York, New York

## Abstract

**Importance:**

Adjuvant stereotactic radiosurgery (SRS) enhances the local control of resected brain metastases (BrM). However, the risks of local failure (LF) and potential for posttreatment adverse radiation effects (PTRE) after early postoperative adjuvant SRS have not yet been established.

**Objective:**

To evaluate whether adjuvant SRS delivered within a median of 14 days after surgery is associated with improved LF without a concomitant increase in PTRE.

**Design, Setting, and Participants:**

This prospective cohort study examines a clinical workflow (RapidRT) that was implemented from 2019 to 2022 to deliver SRS to surgical patients within a median of 14 days, ensuring all patients were treated within 30 days postoperatively. This prospective cohort was compared with a historical cohort (StanRT) of patients with BrM resected between 2013 and 2019 to assess the association of the RapidRT workflow with LF and PTRE. The 2 cohorts were combined to identify optimal SRS timing, with a median follow-up of 3.3 years for survivors.

**Exposure:**

Timing of adjuvant SRS (14, 21, and 30 days postoperatively).

**Main Outcomes and Measures:**

LF and PTRE, according to modified Response Assessment in Neuro-Oncology Brain Metastases criteria.

**Results:**

There were 438 patients (265 [60.5%] female patients; 23 [5.3%] Asian, 27 [6.2%] Black, and 364 [83.1%] White patients) with a mean (SD) age of 62 (13) years; 377 were in the StanRT cohort and 61 in the RapidRT cohort. LF and PTRE rates at 1 year were not significantly different between RapidRT and StanRT cohorts. Timing of SRS was associated with radiographic PTRE. Patients receiving radiation within 14 days had the highest 1-year PTRE rate (18.08%; 95% CI, 8.31%-30.86%), and patients receiving radiation between 22 and 30 days had the lowest 1-year PTRE rate (4.10%; 95% CI, 1.52%-8.73%; *P* = .03). LF rates were highest for patients receiving radiation more than 30 days from surgery (10.65%; 95% CI, 6.90%-15.32%) but comparable for patients receiving radiation within 14 days, between 15 and 21 days, and between 22 and 30 days (≤14 days: 5.12%; 95% CI, 0.86%-15.60%; 15 to ≤21 days: 3.21%; 95% CI, 0.59%-9.99%; 22 to ≤30 days: 6.58%; 95% CI, 3.06%-11.94%; *P* = .20).

**Conclusions and Relevance:**

In this cohort study of adjuvant SRS timing following surgical resection of BrM, the optimal timing for adjuvant SRS appears to be within 22 to 30 days following surgery. The findings of this study suggest that this timing allows for a balanced approach that minimizes the risks associated with LF and PTRE.

## Key Points

**Question:**

Is adjuvant stereotactic radiosurgery (SRS) delivered within 14 days of surgery associated with less local failure (LF) and no greater posttreatment adverse radiation effects (PTRE) among patients with resected brain metastases?

**Findings:**

This cohort study of 438 patients found that adjuvant SRS delivered within 30 days of surgery was associated with less LF; but, if it was delivered within 21 days of surgery, it was associated with more PTRE and, within 14 days of surgery, the most PTRE. Adjuvant SRS delivered within 22 to 30 days was associated with lower rates of PTRE while still maintaining a low LF rate.

**Meaning:**

The findings of this study suggest that the optimal timing for adjuvant SRS of resected brain metastases falls within 22 to 30 days after surgery to minimize both LF and PTRE.

## Teaser

This cohort study evaluates whether adjuvant stereotactic radiosurgery delivered within a median of 14 days after resection of brain metastases is associated with improved local failure without a concomitant increase in posttreatment adverse radiation effects.

## Introduction

Postresection brain metastasis (BrM) cavities typically are treated with adjuvant irradiation given improved local control vs resection alone.^1,2,3^ Conformal stereotactic radiosurgery (SRS) achieves similar local control to whole-brain radiation therapy with fewer cognitive adverse effects and better quality of life.^4,5^ With the introduction of precision targeted cancer therapies and immune checkpoint inhibitors, survival rates for patients with BrMs have improved significantly.^6,7,8,9,10^ These advances have brought the objective of minimizing toxic effects while achieving long-term local control of BrM to the forefront of treatment priorities.

The timing of adjuvant SRS administration is an important factor that affects local control rates following surgery, with prolonged intervals between these paired treatments associated with increased risk of local failure. Several studies have demonstrated that SRS delivered within 1 month of surgery reduces the likelihood of local recurrence vs SRS delivered thereafter.^11,12,13,14,15^ However, concerns have been raised regarding early adjuvant SRS due to potential changes in postoperative cavity size, the potential for impaired wound healing, and challenges in coordinating preradiation imaging and SRS planning.^16,17,18,19^ Previously described multidisciplinary teams have enabled our institution to establish efficient rapid radiotherapy (RapidRT) systems that reduce the time between surgery and radiation to an average of 14 days.^20,21^ Such care delivery systems aim to streamline care, reduce patient burden, and ensure timely systemic cancer-directed treatment resumption for patients’ underlying cancer, which in many cases of advanced disease is also progressive extracranially.

In this retrospective study, we compare local control, posttreatment adverse radiation effects (PTRE, which includes asymptomatic pseudoprogression and radiation necrosis^22^), and wound complications in a prospectively collected registry of patients undergoing a rapid adjuvant SRS (RapidRT) workflow with a historical institutional patient cohort treated using the same dosing and fractionation principles under standard postoperative SRS (StanRT) scheduling. Evaluation of safety and other outcomes of such a paradigm is important in determining whether continuation of RapidRT procedures is warranted for patients requiring complex multidisciplinary care coordination with minimal systemic treatment gaps. This study aimed to evaluate whether adjuvant SRS delivered within a median of 14 days is associated with improved local failure (LF) without concomitant increase in PTRE.

## Methods

All patients in this study received treatment for BrM at Memorial Sloan Kettering Cancer Center between 2013 and 2022. The study was approved, with informed consent waived due to its retrospective nature, by the local institutional review board. We followed the Strengthening the Reporting of Observational Studies in Epidemiology (STROBE) reporting guideline for cohort studies. A prospective database was established as part of a quality improvement initiative to enhance multidisciplinary coordination and expedite adjuvant SRS for resected BrM. This prospective RapidRT cohort consisted of patients evaluated and treated between 2019 and 2022 with a goal of initiating SRS by 2 weeks postoperatively, using a previously described workflow.^20^ A second, historical cohort comprising patients who underwent adjuvant SRS between 2013 and 2019 using a legacy workflow resulting in radiation starting approximately 4 weeks postoperatively was identified retrospectively. The StanRT cohort included any patients with BrM treated with resection plus postoperative SRS within 90 days. Retrospective medical record review identified demographic characteristics, time from surgery to SRS initiation, and dates of local progression, PTRE, complications, death, or last follow-up. Response Assessment in Neuro-Oncology Brain Metastases (RANO-BM) criteria were used to assess recurrence.^23^ PTRE diagnosis was based on radiographic and clinical features of increased enhancement fulfilling RANO-BM criteria for recurrence but without hyperperfusion on magnetic resonance (MR) perfusion imaging, hypermetabolism on brain fluorodeoxyglucose–positron emission tomography (FDG PET) scan, or with spontaneous subsequent regression in the absence of central nervous system (CNS)–directed therapy (eg, reirradiation or CNS-active systemic agent), and regardless of symptom presence or absence. In cases with tissue confirmation, recurrent tumor vs PTRE was based solely on pathology. In the RapidRT group, patients with PTRE were evaluated for corticosteroid dependence, defined as the presence of anatomically referable symptoms requiring steroids beyond the initial 30-day postoperative period, that was not due to other metastases. Radiographic findings were based on board-certified neuroradiologist interpretations of MR imaging (MRI) and computed tomography (CT) studies; these were further reviewed when quantitative features of interest were not commented on. Wound complications were defined as need for antibiotic treatment and/or wound revision following surgery and adjuvant radiation. Patients with all histologic metastasis subtypes were included.

### Radiosurgical Techniques

As previously described,^13^ patients were immobilized for simulation and radiation. For patients who underwent mask-fitting during the operative hospitalization (postoperative day 1-2), any circumferential headwrap bandages were removed; local bandages were maintained. A simulation CT and an MRI obtained within 1 week of simulation were fused for contouring the postoperative cavity and any intact BrM requiring definitive SRS concurrently. Previously irradiated BrM were contoured as objects of avoidance. Postoperative cavities were defined on MRI and/or CT and a 2 to 3 mm uniform expansion margin was used to define the planning target volume (PTV). Treatment plans were generated in Eclipse (Varian Medical Systems). Smaller lesions treated with a single fraction had a 125% to 140% hotspot and a steep dose gradient outside the PTV. Larger cavities treated with hypo-fractionated volumetric modulated arc therapy or intensity-modulated RT had a hotspot in the range 110% to 140% depending on PTV size. All patients were treated on a linear accelerator with 6 MV photons and a multileaf collimator of 1 mm, 2.5 mm, or 5 mm leaves. PTRE management was at the discretion of the treating team.

### Statistical Analysis

Descriptive statistics, such as proportions, means, SDs, and IQRs, were used to characterize the cohorts. Continuous variables were compared across cohorts using the Wilcoxon rank sum test. Categorical variables were compared across cohorts using the χ^2^ and Fisher exact tests as appropriate. Variables that were statistically significantly associated with treatment cohort were considered confounders. Overall survival (OS) was defined from SRS until death for those with events or last follow-up for those who were censored. For the investigation of adjuvant SRS timing with events of interest (OS, local failure, PTRE, and wound complications), all follow-up was used. For the investigation of RapidRT and StanRT cohorts with events of interest, follow-up was truncated at 12 months following SRS so that the cohorts would be comparable. This is because by design the StanRT group had longer follow-up and estimates would be biased without truncation. Furthermore, events within 12 months are of clinical relevance in this patient population across advanced cancers with BrM. Weekly categories of adjuvant SRS timing from 14 days after surgery were defined a priori. OS was compared across groups of interest with the log-rank test. Rates of LF, PTRE, and wound complications were each separately estimated using cumulative incidence and accompanying 95% CIs in the competing risks setting where death was considered a competing event. Cumulative incidence across groups of interest was compared using the Gray test. Multivariable modeling was performed to adjust the association between RapidRT and StanRT cohorts with all events of interest for identified confounders. Multivariable modeling in which the outcome was OS was performed using Cox regression. Multivariable modeling in which the outcome was LF, PTRE, or wound complications was performed using subdistribution hazards modeling in the competing risks setting. Multivariable modeling was not feasible for the association between timing of adjuvant SRS and outcomes due to small event size and a priori defined weekly categories of timing. All statistical tests were 2-sided, and significance was set at *P* < .05. All analyses were performed in SAS version 9.4 (SAS Institute) and R version 4.1.3 (R Foundation for Statistical Computing).

## Results

**Figure 1.**
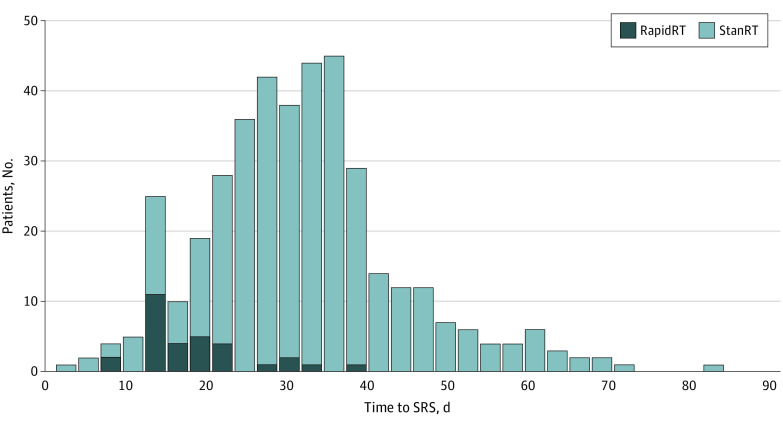
Histogram of Counts for Time to Stereotactic Radiosurgery (SRS) in Rapid Radiotherapy (RapidRT) and Standard RT (StanRT) Cohorts

A total of 438 patients were included, with 377 patients in the StanRT cohort and 61 patients in the RapidRT cohort (Table 1). The mean (SD) age at surgery was 62 (13) years for the entire study population, and there was no significant difference between the cohorts (StanRT, 62 [13] years; RapidRT, 61 [11] years; *P* = .68). There were 265 (60.5%) female patients and 23 (5.3%) Asian, 27 (6.2%) Black, and 364 (83.1%) White patients. Tumor location, extent of resection (gross total vs subtotal), maximum tumor diameter, and number of lesions treated were not significantly different between cohorts. Histology of primary tumor did differ between cohorts, with a higher proportion of breast BrM in the RapidRT cohort vs StanRT cohort (16 patients [26%] vs 54 patients [14%]) and a smaller proportion of lung BrM (10 [16%] vs 129 [34%]) and melanoma BrM (4 [7%] vs 47 [12%]) in the RapidRT cohort vs StanRT cohort (*P* = .04). The median time to radiation after surgery was 14 days (IQR, 13-17 days; range, 2-38 days; 2 patients received SRS 32-38 days due to extenuating circumstances) for the RapidRT patients, which was significantly shorter than the median 32 days (IQR, 25-38 days; range, 7-82 days) for the StanRT cohort (*P* < .001) (Figure 1). Median (IQR) radiation dose did not differ between cohorts (StanRT, 3000 [2700-3000] cGy, RapidRT, 3000 [2700-3000] cGy; *P* = .67). However, there was a significant difference in the number of treatment fractions between the StanRT and RapidRT cohorts; 5 fractions was the most common adjuvant SRS regimen in both cohorts, while the StanRT cohort had a greater rate of single-fraction treatment (32 [9%] vs 0) and the RapidRT cohort had a higher rate of 3-fraction treatments (22 [36%] vs 44 [12%]) (*P* < .001) (Table 1). The median postoperative hospitalization duration was 3 days for both cohorts (*P* = .11). The median follow-up for survivors was 12 months for both StanRT and RapidRT cohorts, and all surviving patients included in this comparison had at least 12 months follow-up except for one who was lost to follow-up. The median (IQR) follow-up for survivors for the associations between adjuvant SRS timing and all outcomes was 3.3 (1.8-4.5) years.

**Table 1.** Demographics by StanRT and RapidRT Cohorts

Characteristic	Patients, No. (%)			*P* value
	Overall (N = 438)	StanRT (n = 377)	RapidRT (n = 61)	
Dichotomized time to SRS				
Time to SRS: ≤30 d	231 (53)	172 (46)	59 (97)	<.001
Time to SRS: >30 d	207 (47)	205 (54)	2 (3.3)	
Age at surgery, y				
Median (range)	62 (20-93)	63 (20-93)	60 (39-79)	.68
Mean (SD)	62 (13)	62 (13)	61 (11)	
Histology				
Lung or NSCLC	139 (32)	129 (34)	10 (16)	.04
Breast	70 (16)	54 (14)	16 (26)	
Melanoma	51 (12)	47 (12)	4 (6.6)	
RCC	19 (4.3)	14 (3.7)	5 (8.2)	
GI	62 (14)	51 (14)	11 (18)	
Sarcoma	16 (3.7)	13 (3.4)	3 (4.9)	
Ovarian	20 (4.6)	16 (4.2)	4 (6.6)	
Uterine or endometrial	13 (3.0)	11 (2.9)	2 (3.3)	
Prostate	10 (2.3)	9 (2.4)	1 (1.6)	
Other or unknown	38 (8.7)	33 (8.8)	5 (8.2)	
Dose, cGy				
Median (range)	3000 (1500-3000)	3000 (1500-3000)	3000 (2500-3000)	.67
Mean (SD)	2811 (322)	2800 (341)	2884 (153)	
Fractions				
1	32 (7.3)	32 (8.5)	0 (0)	<.001
3	66 (15)	44 (12)	22 (36)	
4	2 (0.5)	2 (0.5)	0 (0)	
5	338 (77)	299 (79)	39 (64)	
Lesions treated with surgery and adjuvant SRS and any concurrently treated with definitive SRS				
Median (range)	1.00 (1.00-24.00)	1.00 (1.00-13.00)	1.00 (1.00-24.00)	.69
Mean (SD)	1.93 (1.82)	1.89 (1.50)	2.19 (3.19)	
Unknown	2	0	2	
Maximal tumor diameter, cm				
Median (range)	3.10 (0.80-7.60)	3.10 (0.80-7.60)	3.30 (1.70-6.60)	.36
Mean (SD)	3.23 (1.18)	3.21 (1.19)	3.35 (1.07)	
Unknown	2	0	2	
LOS from surgery, d				
Median (range)	3.00 (0.00-28.00)	3.00 (0.00-26.00)	3.00 (1.00-28.00)	.11
Mean (SD)	3.92 (3.40)	3.88 (3.13)	4.16 (4.77)	
Time to SRS, d				
Median (range)	30 (2-82)	32 (7-82)	14 (2-38)	<.001
Mean (SD)	31 (13)	33 (12)	16 (6)	
Side				
Left	213 (49)	182 (48)	31 (51)	.17
Midline	5 (1.1)	3 (0.8)	2 (3.3)	
Right	220 (50)	192 (51)	28 (46)	
Location of index resected brain metastasis				
Cerebellar	85 (19)	72 (19)	13 (21)	.66
Frontal	145 (33)	127 (34)	18 (30)	
Multiple	10 (2.3)	7 (1.9)	3 (4.9)	
Occipital	52 (12)	46 (12)	6 (9.8)	
Parietal	97 (22)	84 (22)	13 (21)	
Temporal	49 (11)	41 (11)	8 (13)	

Abbreviations: GI, gastrointestinal; LOS, length of stay; NSCLC, non–small cell lung cancer; RapidRT, rapid radiotherapy; RCC, renal cell carcinoma; SRS, stereotactic radiosurgery; StanRT, standard radiotherapy.

### Clinical Outcomes by Treatment Cohort

**Figure 2.**
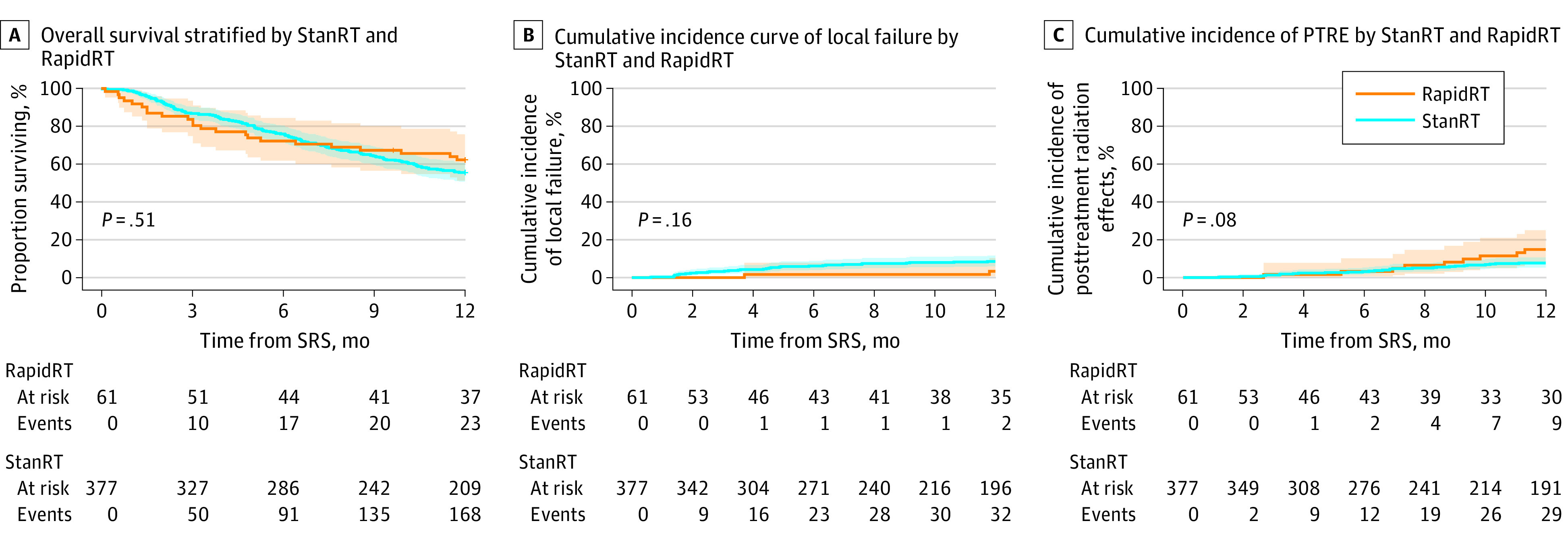
Overall Survival, Local Failure, and Posttreatment Adverse Radiation Effects (PTRE) in Rapid Radiotherapy (RapidRT) and Standard RT (StanRT) Cohorts

Median OS was not reached in either cohort with 1-year survival rate of 55.44% (95% CI, 50.64%-60.69%) for StanRT and 62.17% (95% CI, 51.09%-75.65%) for RapidRT (log-rank *P* = .51) (Figure 2A). LF rates at 1 year were not significantly different (Gray test *P* = .20), with failure rates of 8.49% (95% CI, 5.95%-11.58%) for StanRT and 3.32% (95% CI, 0.61%-10.33%) for RapidRT (Figure 2B).

Cumulative incidence of PTRE at 1 year was 7.69% (95% CI, 5.28%-10.67%) for StanRT and 14.89% (95% CI, 7.25%-25.12%) for RapidRT, which did not reach statistical significance (Gray test *P* = .08) (Figure 2C). Overall, 69% of patients with PTRE (6 of 9) in the RapidRT cohort were steroid-dependent at 12 months. Wound complications were rare in both StanRT and RapidRT cohorts, with 1-year rates of 1.06% (95% CI, 0.36%-2.55%) and 1.64% (95% CI, 0.13%-7.81%), respectively, and did not demonstrate statistical difference (Gray test *P* = .70). There were no differences in any outcomes by treatment cohort even after adjustment for confounders (histology and number of fractions) in multivariable models.

### Clinical Outcomes Stratified by Time to Radiation

**Figure 3.**
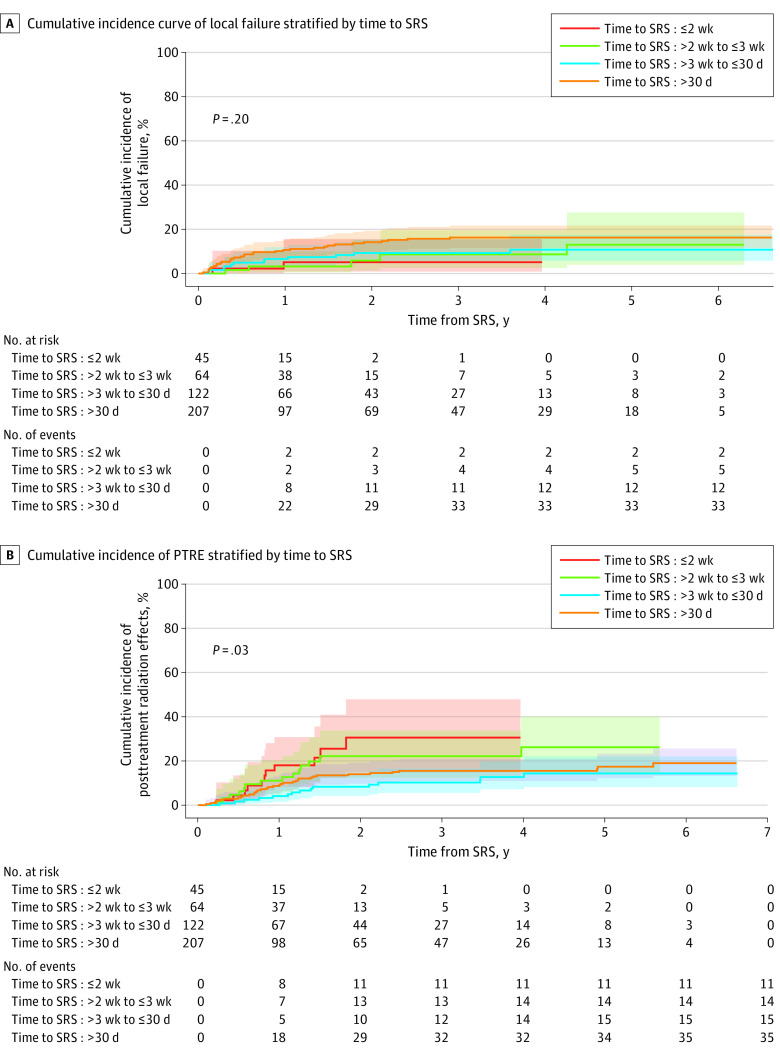
Incidence of Local Failure and Posttreatment Radiation Effects (PTRE) by Time to Stereotactic Radiosurgery (SRS)

Given the wide range of time to administration of radiation in the StanRT cohort, including many patients treated within 14 to 30 days, we subsequently unified the patient population and extended follow-up to evaluate the association of time to radiation with outcomes in all 438 patients. Patients from both the StanRT and RapidRT cohorts were stratified by time to radiation at a priori defined clinical cut points of interest: 14 days or fewer, 15 to 21 days, 22 to 30 days, and 31 days or more. OS at 1 year did not significantly differ by time point stratification. LF rates were highest for patients receiving radiation more than 30 days from surgery (10.65%; 95% CI, 6.90%-15.32%) but comparable for patients receiving radiation within 14 days, between 15 and 21 days, and between 22 and 30 days (≤14 days: 5.12%; 95% CI, 0.86%-15.60%; 15 to ≤21 days: 3.21%, 95% CI, 0.59%-9.99%; 22 to ≤30 days: 6.58%, 95% CI, 3.06%-11.94%; Gray test *P* = .20). LF and clinical cut points of radiation timing are shown in Table 2 and Figure 3A.

**Table 2.** Yearly Event Rates Stratified by Time to SRS

Time to SRS, d	Patients, No. (N = 438)	PTRE cumulative incidence, % (95% CI)				*P* value	LF cumulative incidence, % (95% CI)				*P* value
		Events, No. (n = 75)	1-y	2-y	3-y		Events, No. (n = 52)	1-y	2-y	3-y	
≤14	45	11	18.08 (8.31-30.86)	30.66 (14.88-48.01)	30.66 (14.88-48.01)	.03	2	5.12 (0.86-15.60)	5.12 (0.86-15.60)	5.12 (0.86-15.60)	.20
>14 to ≤21	64	14	11.12 (4.84-20.33)	22.23 (12.37-33.88)	22.23 (12.37-33.88)		5	3.21 (0.59-9.99)	5.85 (1.40-15.14)	8.67 (2.55-19.60)	
>21 to ≤30	122	15	4.10 (1.52-8.73)	8.35 (4.25-14.21)	10.27 (5.57-16.63)		12	6.58 (3.06-11.94)	9.30 (4.89-15.41)	9.30 (4.89-15.41)	
>30	207	35	8.70 (5.35-13.04)	14.06 (9.71-19.19)	15.56 (10.97-20.88)		33	10.65 (6.90-15.32)	14.20 (9.81-19.37)	16.30 (11.57-21.74)	

Abbreviations: LF, local failure; PTRE, posttreatment adverse radiation effects; SRS, stereotactic radiosurgery.

PTRE rates were associated with time to radiation (Gray test *P* = .03) (Table 2 and Figure 3B). Patients receiving radiation within 14 days demonstrated the highest 1-year PTRE rate of 18.08% (95%CI, 8.31%-30.86%), and patients receiving radiation between 22 and 30 days demonstrated the lowest 1-year PTRE rate (4.10%; 95% CI, 1.52%-8.73%).

There were very few wound complications overall and no wound complications within 14 days. There were no significant differences in wound complication rates for patients treated within 21 days (1-year cumulative incidence 0.92%; 95% CI, 0.08%-4.56%) and those treated after 21 days (1.22%; 95% CI, 0.41%-2.92%) (Gray test *P* = .73).

## Discussion

Adjuvant SRS is the contemporary standard of care following BrM resection.^1,2,3,24,25,26^ Local control rates with adjuvant SRS ranges from 50% to 94% at 1 year,^13,24,25,27,28,29,30,31,32^ with efficacy in the large multi-institutional pivotal studies in the range of 70% to 75%. Substantial attention has been devoted to optimizing efficacy and reducing potential causes of institutional variability, with studies attempting to clarify the impacts of dosing, fractionation, and margins on outcome. Recent work has highlighted the importance of variable SRS timing after surgery on local control.^11,12,13,14,15,33^ These retrospective studies have highlighted improved outcomes with adjuvant SRS delivered before 3 to 6 weeks following surgery. The current study used a prospective registry of ultra-early postoperative SRS (RapidRT) and a large institutional cohort of patients treated with a similar radiosurgical paradigm using single- and hypo-fractionated SRS to identify whether early radiation started at a median of 14 days could be beneficial. We found a consistent local control benefit associated with radiation initiation within 30 days of surgery. However, PTRE rates suggested an association with time from surgery to radiation such that patients receiving radiation within 14 days exhibited significantly higher 1-year PTRE rates (approximately 18%), while the lowest PTRE rate was observed in patients starting radiation 22 to 30 days after surgery. Although concerns regarding cavity dynamics with early radiation have been raised, empirical effects of these findings on clinical outcomes remain unclear. However, without formal analyses, we did not observe any obvious change in cavity size when designing RT plans in the RapidRT cohort vs the StanRT cohort. Additionally, we did not observe any obvious cavity discrepancies on cone beam CT taken before treatment daily in comparison with the planning CT cavities in either group. This report demonstrates the empirical importance in balancing the benefits of early radiation on local control and the risk of PTRE, with the optimal timing seeming to occur between 22 and 30 days.

The excellent, 1-year 97% local control rate for the RapidRT cohort and patients receiving radiation within the third week after surgery (local control, 93%) or within 30 days (local control, 93%-97%) compares favorably with the literature. While the local control comparison of the RapidRT and StanRT cohorts did not achieve statistical difference, this was likely confounded by the significant proportion of patients (46%) who received radiation within 30 days of surgery in the StanRT group. Once the analyses were stratified by time to radiation within the larger combined cohort, this association became clearer. It is also important to note that receipt of adjuvant radiation early can improve patient burden by reducing visits, scan requirements, and time to resumption of systemic cancer-directed therapy.^14,15,20^ Importantly, OS was not different between RapidRT and StanRT cohorts or between cohorts stratified by time to radiation at any time point. This suggests that other retrospective studies that demonstrated poor survival in patients receiving ultra-early irradiation (<22-23 days)^14,15^ may have been confounded by other potential correlates, such as rapidly progressive extracranial extent of disease.^34^

Importantly, no difference in wound complications was seen across RapidRT vs StanRT cohorts or when stratified by time to radiation. While early radiation has long been considered a concern for wound healing,^16^ little empirical data actually exist in the era of highly conformal SRS to demonstrate any effect of radiation timing on wound healing. Such wound healing concerns have in part been based on historical experiences with whole-brain treatment plans that include skin dose and appear to not be relevant with SRS, which spares any significant dose to the skin. This topic has also been of interest in the postoperative spine metastasis literature, with no clear wound harm identified with early image-guided radiation.^35,36^ Our study provides prospective and retrospective evidence that SRS, even within a median of 14 days after surgery, was associated with no significant wound-healing risk.

PTRE, however, remains a salient complication of SRS. The cumulative 1-year incidence of PTRE for patients initiating radiation within 14 days was 18%, similar to the range of some prior studies of adjuvant single-fraction and hypo-fractionated SRS (5%-17%),^28,29,37,38^ but it was more than 4 times that of patients in the current study receiving radiation between 22 and 30 days after surgery (4.1%). However, not all patients with PTRE were symptomatic, and only 69% of the RapidRT cohort patients with PTRE were steroid-dependent. However, the association between radiation at the earliest studied time points and PTRE does suggest a potential risk of expediting adjuvant SRS. The increased PTRE rate could be due to previously described early cavity volumetric changes, with earlier radiation planning ultimately covering more brain parenchyma if cavities temporarily contracted between postoperative simulation and RT start.^18,19,31,39^ Alternatively, the increased PTRE rate could also be related to treating tissue that is still devascularized and thus oxygen-depleted postoperatively and unable to repair sublethal radiation damage. Finally, it is also well-recognized that certain systemic therapies elevate the risk of PTRE, and given that the RapidRT cohort was treated more recently, some of these patients were likely receiving newer agents such as antibody drug conjugates, which have recently been reported to elevate the risk of PTRE, including radiation necrosis.^40,41^

### Limitations

This study has limitations. The retrospective nature of the study results in potential confounding biases between groups, which may affect survival, LF, or PTRE rates, although we did not see measurable changes in estimates after adjustment for confounders, such as histology and radiation fractions.^42,43^ While the follow-up of 12 months for the association of StanRT and RapidRT cohort with outcomes is limited, this is a reasonable proxy given the limited OS of this patient population. Furthermore, the median follow-up of 3.3 years for the adjuvant SRS timing association with outcomes compares favorably with the literature.^44^ The study may not be powered to demonstrate differences in rare events, such as wound complications; however, such studies are not generally feasible given the cohort sizes that would be required. Finally, given the focus on the association between radiation timing and treated-lesion local control rather than out-of-field outcomes and the dynamic nature of drug development and approvals, we do not report systemic treatment or palliative histories, which are important determinants of survival and in some cases overall CNS (if not necessarily resection-field) outcomes. Importantly, an increasing menu of highly CNS-active targeted therapies including for *EGFR-*variant and *ALK*-rearranged malignant neoplasms allows a growing proportion of patients to avoid metastasectomy and/or brain radiation. Given low event rates, wound healing may also be impacted by newer agents without well-defined perioperative risks. As such, further study is required to optimize the timing and sequencing of both systemic and CNS-directed local treatments.

## Conclusions

In this cohort study, the timing of administering adjuvant SRS following surgical resection of BrM was significantly associated with local control and PTRE. Our findings suggest that achieving the optimal timing for adjuvant SRS, specifically within 22 to 30 days following surgery, allows for a balanced approach that is associated with minimized risks of LF and PTRE.

## Notes

Supplement.
Data Sharing Statement
