# Supplementary material for: Outcomes Following Early Postoperative Adjuvant Radiosurgery for Brain Metastases
Source: JAMA Netw Open. 2023 Oct 31;6(10):e2340654. doi: 10.1001/jamanetworkopen.2023.40654 (PMC10618851; doi:10.1001/jamanetworkopen.2023.40654)
Supplement: Supplement. — Data Sharing Statement [file jamanetwopen-e2340654-s001.pdf]

Data Sharing Statement

Bander. Outcomes Following Early Postoperative Adjuvant Radiosurgery for Brain Metastases. *JAMA Netw Open*. Published October 31, 2023. doi:10.1001/jamanetworkopen.2023.40654

Data
Data available: No
